# Quantifying Viscosity of Semisolid Submicrometer and Nanosized Individual Aerosols Using Atomic Force Microscopy with Varying Cantilever Velocity

**DOI:** 10.1021/acs.analchem.6c02398

**Published:** 2026-10-01

**Authors:** Ruwini A. Weerasinghe, Chamika K. Madawala, Alexei V. Tivanski

**Affiliations:** Department of Chemistry, 4083 University of Iowa, Iowa City, Iowa 52242, United States

## Abstract

Atmospheric aerosol viscosity governs diffusion time scales of reactive gases into aerosols, thereby influencing gas uptake, heterogeneous chemistry, and atmospheric aging. However, direct viscosity measurements of individual semisolid aerosols have been restricted to a relatively high viscosity range and submicrometer size. Herein, a new atomic force microscopy-based nanorheological experimental approach is presented that utilizes force–indentation distance and indentation rate measurements at varying cantilever velocities to quantify the viscosity of individual semisolid aerosols as a function of relative humidity (RH). Using glucose and sucrose as model systems, we demonstrate accurate single particle viscosity quantification spanning ∼10^2^·^5^ to 10^7^ Pa s under atmospherically relevant RH conditions. We establish an iterative cantilever velocity framework that enables the determination of the appropriate velocity required for accurate viscosity characterization of aerosols without prior knowledge of their composition. Additionally, we demonstrate that the method enables accurate viscosity quantification of individual sub-100 nm aerosols. We anticipate that future viscosity measurements of atmospheric aerosols from various sources at different RHs will provide valuable insights into aerosol viscosity variability and reveal the extent of particle-to-particle heterogeneity.

## Graphical abstract

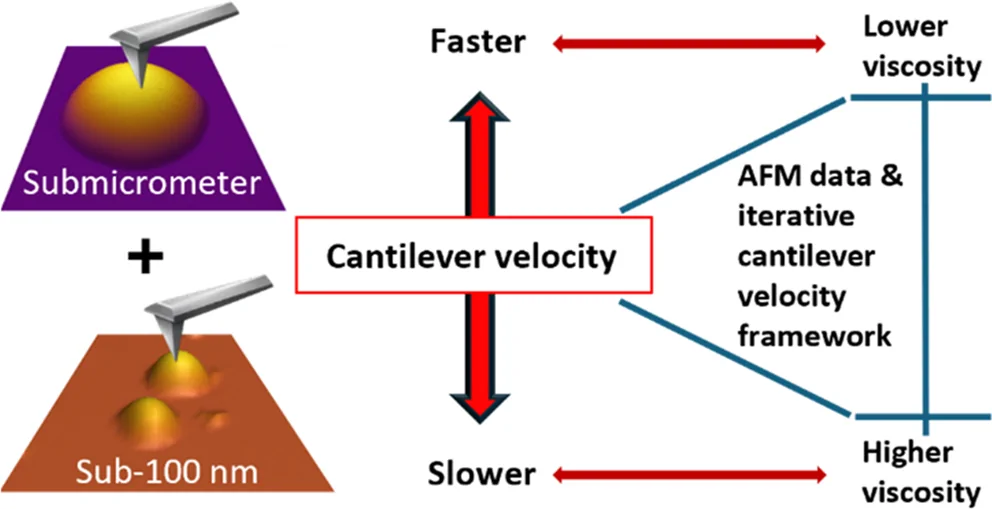

## Introduction

Atmospheric aerosols are air-suspended solid, semisolid, or liquid particles with highly variable composition, mixing states, and sizes.^1–3^ They are typically categorized by size categories as supermicrometer, submicrometer, and sub-100 nm with a significant majority within submicrometer and sub-100 nm size ranges.^4–7^ Atmospheric aerosols play a major role in regulating the Earth’s radiative forcing and various atmospheric processes via direct and indirect aerosol effects.^2,4,8–10^ The direct effect relates to scattering and absorbing of solar radiation, while the indirect effect accounts for their role as cloud condensation nuclei (CCN) or ice nucleating particles (INPs).^2,11–15^ The overall effect depends on the source and type of aerosols, and further varies depending on the aerosols’ size, morphology, mixing states, composition, phase state, and viscosity, all of which in turn depend on varying atmospheric relative humidity (RH) and atmospheric aging.^2,16–18^ Thus, to be able to fully quantify and ultimately predict the overall effect of atmospheric aerosols on climate and environment, these properties need to be characterized. In particular, atmospheric aerosols’ phase state (solid, semisolid, and liquid)^19^ and corresponding viscosity ranges (>10^12^ Pa s for solids, 10^12^ to 10^2^ Pa s for semisolids, and <10^2^ Pa s for liquids)^20^ can modulate diffusion time scales of various atmospheric gases within aerosols, in turn affecting their atmospheric aging and their ability to act as efficient CCN or INPs.^20–23^ Furthermore, since a substantial fraction of atmospheric aerosols is in semisolid state,^19,24,25^ coupled with almost 10 orders of magnitude viscosity range of semisolid aerosols (10^12^ to 10^2^ Pa s), the diffusion time scales of atmospheric gases in or out of the semisolid aerosols can range from years to less than a second depending on their viscosity,^17,20^ thus significantly impacting their atmospheric aging process and ultimately their climate-relevant effects.^20,26^ Hence, direct viscosity measurements of semisolid aerosols are critical and must be conducted under varying RH conditions. Additionally, such measurements should focus on sub-100 nm aerosols, which are highly abundant, as well as submicrometer aerosols, which have an extended atmospheric lifetime.^27,28^ Noteworthy, direct individual aerosol viscosity quantification is also needed, as extrapolating viscosity measurements from supermicrometer to submicrometer or sub-100 nm aerosols can be unreliable due to their size-dependent chemical composition.^18^ Furthermore, single particle viscosity measurements uniquely offer an opportunity to assess the extent of particle-to-particle variability for measured viscosity within a subpopulation of aerosols (e.g., within a particular size range from the same aerosol source).^16,18,24,25^

Recently, we reported how atomic force microscopy (AFM) force–indentation distance measurements coupled with the Kelvin–Voigt viscoelastic model can be used to directly quantify the viscosity within ∼10^4^–10^7^ Pa s range of individual semisolid submicrometer aerosols as a function of RH and without prior knowledge of aerosol’s composition.^29^ However, the method had a significant limitation, where once expected aerosol viscosity approaches ∼10^4^ Pa s at a particular elevated RH, AFM measured viscosity values became relatively insensitive to further increases in RH, resulting in an apparent plateau at ∼10^4^ Pa s. As such apparent inability to quantify aerosol viscosities below ∼10^4^ Pa s significantly limits utility of the AFM method, the present study focuses on developing new experimental approaches that enable single particle viscosity quantification at the lower viscosity range, which in turn expected to more accurately describe atmospheric aging of aerosols under varying conditions (e.g., rapid changes in atmospheric RH and corresponding diffusion times of water molecules within the aerosol with low semisolid viscosity). We noted that the previous AFM viscosity method was based on utilizing a relatively low and fixed cantilever velocity value of ∼0.7 μm/s. However, as aerosol viscosity becomes less than ∼10^4^ Pa s, we hypothesized that such a relatively low cantilever velocity is unable to accurately measure the rate of indentation, ultimately leading to limitations of the original method to quantify viscosity below ∼10^4^ Pa s. Our hypothesis is based on previously published studies related to AFM nanorheology, where the viscoelastic response of soft biological cells to AFM indentation was found to critically depend on the time scale (frequency) over which the indentation is applied, implying that the measured viscoelastic behavior reflects the interplay between elastic and viscous contributions at a particular indentation rate.^30–32^ Since the time scale of the indentation can be modulated by cantilever velocity, the present work aimed to develop a new experimental approach based on AFM force and indentation rate measurements at varying cantilever velocities that we hypothesized would enable single particle viscosity quantifications below ∼10^4^ Pa s.

Here, we utilize AFM force–indentation distance and rate of indentation measurements using various cantilever velocities as a function of RH on two model systems: individual glucose and sucrose particles. The model systems were selected since these are present in sea spray aerosols and outdoor environmental films,^33–36^ and literature data exist that establishes a direct RH-viscosity relationship for each model system,^37^ thus enabling a direct comparison between AFM-based viscosity measurements with expected viscosity at a particular RH. We show how the new cantilever-dependent AFM measurements enable single particle viscosity quantification within the viscosity range of ∼10^2.5^–10^7^ Pa s, consequently extending the lower viscosity range amendable for quantification by almost 2 orders of magnitude. Furthermore, we introduce an iterative cantilever velocity classification framework that allows identification of the appropriate cantilever velocity needed for accurate viscosity quantification of an individual aerosol at a particular RH. Finally, we extended AFM-based single particle viscosity measurements to individual sub-100 nm particles, where we show the method is capable of accurately quantifying viscosity of sub-100 nm aerosols with heights greater than ∼20 nm.

## Experimental Section

### Particle Generation

Glucose and sucrose were purchased from Sigma-Aldrich, as reagent grade 99.99% purity. Both chemicals were used without additional purification, and they were dissolved in deionized water (resistivity of **∼**22 MΩ·cm) at 100 mM molar concentration. Glucose and sucrose aerosols were generated using a constant output atomizer (TSI, Inc., Model 3076) from 100 mM aqueous solutions. The aerosol stream was mixed with humidified N_2_ gas at a constant rate of 30 L/min to achieve ∼80% relative humidity (RH) in the mixing chamber and then a Micro Orifice Uniform Deposit Impactor (MOUDI; MSP, Inc., Model 110) was used to deposit the aerosols by impaction in a size-resolved manner. Aerosols were deposited on hydrophobically coated (Rain-X) silicon wafers (Ted Pella Inc.) placed on the MOUDI stages 7, 9, and 10, corresponding to aerodynamic diameters of 0.33–0.56 μm, 0.10–0.18 μm, and 0.055–0.10 μm, respectively. For submicrometer glucose and sucrose particles study, aerosols were collected using MOUDI stage 7, whereas for glucose nanoparticles study, aerosols were collected using MOUDI stages 9 and 10. The substrate-deposited glucose or sucrose particles were stored in clean Petri dishes and kept inside a laminar flow hood (NuAire, Inc., NU-425–400) at room temperature (20–25 °C) and ambient pressure at 20–25% RH, and AFM experiments were performed on the same or following day after substrate-depositing particles.^29,38^

### Atomic Force Microscopy (AFM) Imaging, Force–Indentation Distance and Rate of indentation Measurements

A molecular force probe 3D AFM (Asylum Research, Santa Barbara, CA) was used for AFM height imaging and force measurements at room temperature (20–25 °C) and varying RH ranging from **∼**20% to 60% using a custom-made humidity cell.^39^ The equilibration time after each change in RH was approximately 10–15 min to ensure substrate-deposited particles are in thermodynamic equilibrium with surrounding water vapor at a particular RH.^7,19,29,39^ silicon nitride AFM probes (MikroMasch, Model CSC37) with a nominal spring constant of 0.35 N/m and tip radius of curvature of ∼10 nm were used for both imaging and force measurements. Actual spring constants were quantified using the build-in thermal noise method.^40^ All samples were first imaged in an AC (taping) mode to locate individual particles at ∼30% RH. AFM force measurements were next performed in contact mode using varying cantilever velocities by measuring at a particular cantilever velocity force acting on the AFM tip as a function of vertical piezo displacement (i.e., force plots) as the tip moved toward and away from an approximate center of a particle. The force plots were then converted to force versus indentation distance and indentation versus time profiles, allowing quantification of the rate of indentation and subsequent viscoelastic analysis toward quantification of single particle viscosity.

### Viscosity of Individual Submicrometer Glucose and Sucrose Particles as a Function of RH and Cantilever Velocity

To quantify viscosity of aerosols as a function of RH, typically 3–4 force plots were collected for each particle at a particular RH ranging from ∼20% to 60%, using two selected cantilever velocities of 0.7 μm/s and 13.6 μm/s. For each sample at a given RH, the approach data in the contact region, where the tip is indenting into the particle, was used to determine the particle viscosity at corresponding RH. Viscosity values were determined for each aerosol model system (i.e., glucose and sucrose) at a particular RH, where each value reported as an average of repeated 3–4 force plots and one standard deviation. The RH-viscosity relationships for glucose and sucrose were taken from Song et al.^37^ and used for comparison with the AFM-based measurements. To examine how the cantilever velocity impacts viscosity quantification, force plots were also collected at multiple cantilever velocities (0.7, 3.0, 5.0, 6.9, 10.0, 12.1, 13.7, 15.8, 18.0, and 19.7 μm/s) and at two specific RH values: 40% and 50% for glucose, and 50% and 56% for sucrose.

### Application of AFM Viscoelastic Method for Sub-100 nm Individual Semisolid Aerosols

To extend the AFM method of viscosity quantification to sub-100 nm aerosols, viscosities of individual glucose nanoparticles were studied as a function of their size. A total of 14 individual nanoparticles with heights ranging from 20 to 100 nm were studied. The height of individual nanoparticles was determined from AFM height images collected at ∼30% RH.^7,19,41^ For viscosity quantification, for each nanoparticle, typically 3 repeated force plots were collected at an approximate particle center at 35% RH with 0.7 μm/s cantilever velocity, and at 50% RH with 13.6 μm/s cantilever velocity.

## Results and Discussion

Previously, we have successfully developed AFM-based viscoelastic method to accurately quantify viscosity of individual submicrometer particles as a function of RH within the viscosity range of ∼10^4^–10^7^ Pa s.^29^ The viscoelastic expression developed for quantification of single particle viscosity was derived based on the three-dimensional Kelvin–Voigt viscoelastic framework and Hertzian contact mechanics^29^ and can be described by eq 1

1 $$F\left(\mathrm{RH},I,İ\right)=\frac{4}{3}\frac{E\left(\mathrm{RH}\right)}{1-\nu^{2}}\sqrt{R}{I\left(\mathrm{RH}\right)}^{3/2}+\frac{6}{1-\nu^{2}}\sqrt{R}\eta \left(\mathrm{RH}\right)İ\left(\mathrm{RH}\right){I\left(\mathrm{RH}\right)}^{1/2}-F_{\mathrm{adh}}\left(\mathrm{RH}\right)$$

where *F*(RH,*I*,*İ*) is the force measured over a particle at a particular RH and at indentation distance *I* at time *t*, *İ* is the first derivative of indentation distance with respect to time (i.e., rate of indentation), *E* and η are Young’s modulus and viscosity of the particle, respectively, ν is the particle Poisson’s ratio, and *F*_adh_ is the adhesion force between AFM tip and particle and *R* is the AFM tip radius of curvature..^29^ The variables *E*, *I*, *İ F*_adh_ and η are functions of RH. To quantify single particle viscosity at a particular RH, corresponding force–indentation data collected on the particle is fit using eq 1 to determine *E*, *F*_adh_, and product of η*İ*. The maximum indentation is generally limited to the tip radius of curvature (10 nm here) since the model assumes indenting AFM tip shape as a sphere with the radius of *R* corresponding to the tip radius of curvature.^29^ The Poisson’s ratio of glucose and sucrose particles studied here is assumed to be RH-independent and equal to 0.3 to minimize fitting parameters, consistent with our previous work.^29,42,43^ The viscosity results presented below were assessed with respect to how sensitive the determined viscosity value to varying Poisson’s ratios, where viscosity changes were less than 3% for the Poisson’s ratio ranging between 0.3 and 0.5. Noteworthy, the fit using eq 1 yields the product of viscosity and rate of indentation, which cannot be decoupled using the force–indentation data alone. Instead, the viscosity η can be quantified from the product of viscosity and the rate of indentation, which is determined by performing a linear fit to the indentation distance versus time data, yielding the slope, assumed to be the rate of indentation. While the method was shown to accurately quantify viscosities of individual submicrometer particles within the 10^4^–10^7^ Pa s range, one of the objectives of the present study was to extend its applicability to viscosities below 10^4^ Pa s, as less viscous aerosols exhibit much faster diffusion time scales of various reactive atmospheric gases that have significant implications for their atmospheric aging and, in turn, impact their climate-related effects.^20,29^ To achieve this objective, we initially followed the same experimental procedure as outlined above, while now also expanding the studied RH range toward expected aerosols viscosity values below 10^4^ Pa s.

Figure 1A shows a representative AFM 3D height image of an individual submicrometer glucose particle at ∼30% RH displaying rounded morphology with a height of ca. 200 nm, a base size of ca. 1100 nm, and the AFM determined contact angle between the particle and the underlying substrate of ∼66° (Figure S1). The red cross corresponds to the approximate center of the particle where AFM force measurements were performed. By following the previously established method outlined above, Figure 1B shows AFM quantified viscosity of an individual submicrometer glucose particle as a function of RH (red dots are data) over 20%–60% RH range. Here, dots are average viscosity values based on 3–4 individual force–indentation measurements, and error bars represent one standard deviation of the mean. The black dashed lines indicate expected viscosity values reported by Song et al.,^37^ while the pink shaded area represents ±10% uncertainty for the expected values. As can be seen from Figure 1B, data closely follow the expected reduction in the viscosity with increasing RH within the viscosity range of ∼10^4^ to 10^7^ Pa s, consistent with the previous report.^29^ A continuous decrease in the particle viscosity with increasing RH is expected, since elevated RH results in increased water uptake, which in turn decreases solute concentration, resulting in a less viscous particle.^44^ However, measured viscosity values at and above 50% RH (indicated by red oval in Figure 1B) show clear deviation from the expected reduction in the viscosity trend, where an apparent plateau in the determined viscosity values is observed. We next discuss the origin of this deviation and then demonstrate how we modified our experimental approach to develop a new experimental strategy toward overcoming this deviation and enabling accurate quantification of single particle viscosity with values below 10^4^ Pa s.

**Figure 1:**
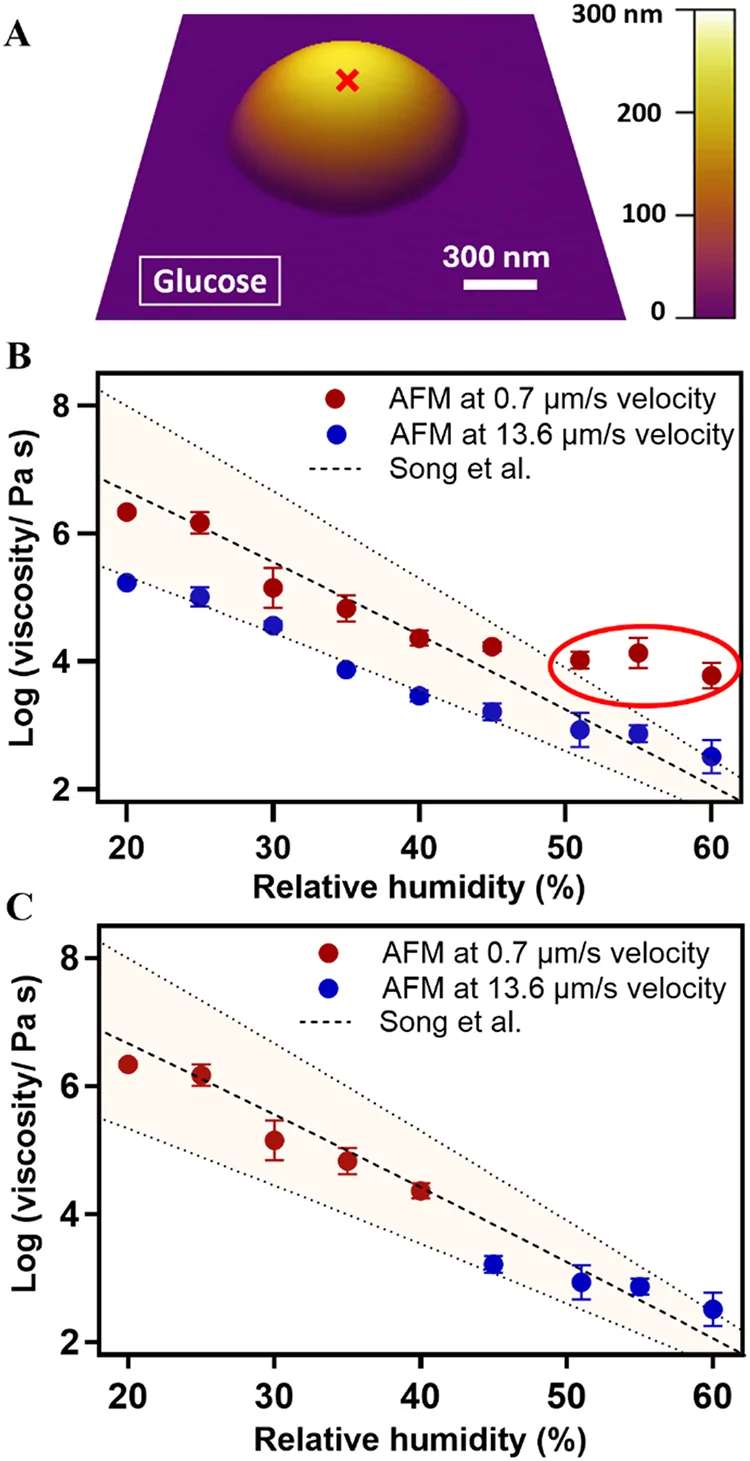
(A) Representative AFM 3D height image of a single glucose particle at ∼30% RH displaying rounded morphology (ca. 200 nm height and ca. 1100 nm base size). The red cross indicates where force measurements were performed. (B) Average AFM viscosity of an individual submicrometer glucose particle as a function of RH at two cantilever velocities. (red, 0.7 μm/s and blue, 13.6 μm/s) along with the expected values from Song et al.^37^ (black dashed line), where the pink shaded area represents ±10% uncertainty. Red oval indicates the data points that deviate beyond this range. (C) Combined average AFM viscosity data selected using the iterative cantilever velocity framework, showing improved agreement with literature values across the full RH range. Error bars represent one standard deviation.

To better understand why AFM viscosity results on glucose appear to deviate at RH above 50%, we compared AFM force data collected on a single glucose particle at 40% RH, where AFM yields an accurate viscosity value, and 50% RH, where deviations begin to emerge. The force–indentation data at both 40% and 50% RH fit well with the viscoelastic model (Figure 2A), confirming that the data follow the model described by eq 1. As noted above, the fit does not directly determine viscosity, instead it determines the product of η*İ*. Given that the data follows the model, we hypothesized that the origin of deviation observed at and above 50% RH for the glucose particle is due to inaccurate determination of the rate of indentation at elevated RH. Figure 2B shows the indentation distance at the contact with the particle surface (i.e., positive indentation distance) versus time at 40% and 50% RH. The zero time corresponds to zero indentation distance when the AFM tip just contacts the particle surface. Each indentation distance versus time plot is clearly linear and thus was fit to a straight line (solid lines are fit), yielding the slope (*İ*, rate of indentation) of 955 ± 6 nm/s (*R* ^2^ = 0.9993) and 967 ± 6 nm/s (*R* ^2^ = 0.9991) for 40% and 50% RH, respectively. It is noticeable that the determined rate of indentation does not appreciably change between 40% and 50% RH. This is opposite to our expectation, since a particle at elevated RH is expected to exhibit greater water uptake. This reduces the solute concentration and, consequently lowers particle viscosity, which should increase the rate of indentation with increasing RH. Thus, the behavior of the rate of indentation versus RH was next examined in more detail.

**Figure 2:**
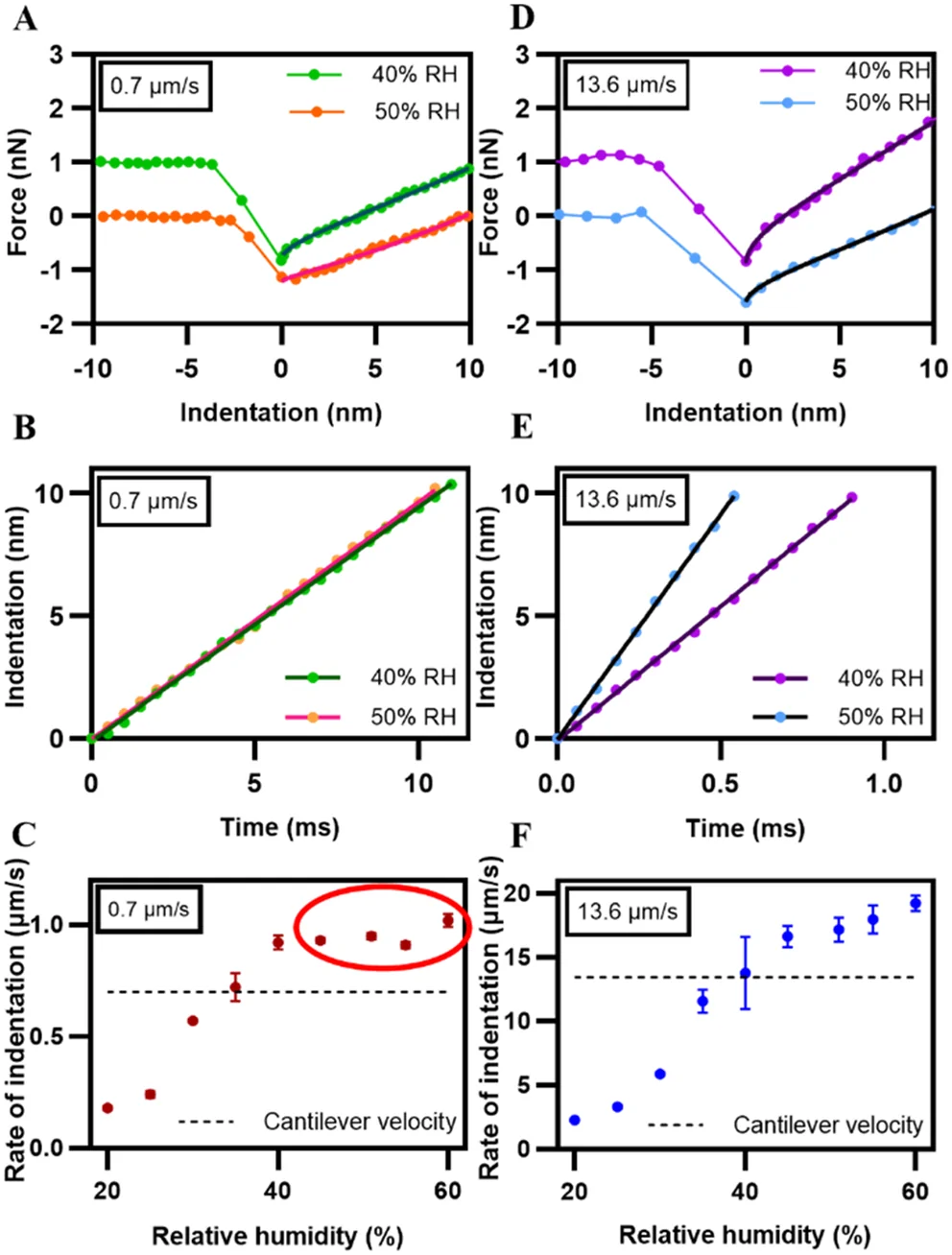
AFM measurements on an individual submicrometer glucose particle. (A, D) Force vs indentation distance curves at 0.7 μm/s and 13.6 μm/s cantilever velocities, respectively, with fits (solid lines) to eq 1 in the contact region; selected RH values are shown (40% and 50% in dots). The force data at 40% RH were offset by 1 nN for clarity. Only approach to the particle surface data is shown. (B, E) Indentation distance vs time at 0.7 μm/s and 13.6 μm/s velocities, respectively, with linear fits used to quantify the first derivative of indentation distance with respect to time. (C, F) Average rate of indentation vs RH for 0.7 μm s^–1^ (red) and 13.6 μm s^–1^ (blue). Dashed lines indicate relevant cantilever velocities. Red oval indicates data points deviating from the expected increasing trend.

Figure 2C shows the AFM determined rate of indentation *İ* versus RH, where the rate increases steadily with increasing RH until ∼40%, where a plateau becomes apparent at higher RH. Since the rate of indentation is expected to continuously increase with increasing RH, this result clearly indicates that our determined rates of indentation at and above ∼45% RH are lower than they should be, leading to an overestimation of the viscosity value. To better understand this observation, we first noted that the plateau rate of indentation value is greater than the cantilever velocity of 0.7 μm/s used to collect this data. We thus hypothesized that such a relatively slow cantilever velocity may limit the accurate determination of the rate of indentation, which in turn leads to overestimation of viscosity at elevated RH, where expected viscosity values are below ∼10^4^ Pa s. To test this hypothesis, we repeated the experiments outlined above at 40% and 50% RH, using a higher cantilever velocity of 13.6 μm/s (ca. 20-fold increase). Figure 2D shows the force–indentation data at 40% and 50% RH collected with 13.6 μm/s velocity, where each data fits well using eq 1, confirming the data follows the model at much higher cantilever velocity. Significantly, Figure 2E shows the indentation distance versus time at 40% and 50% RH collected at 13.6 μm/s cantilever velocity, where a clear increase in the slope is observed with RH increase, as expected. Specifically, the fitted *İ* values are 10,970 ± 80 nm/s at 40% RH (*R* ^2^ = 0.9993) and 18,380 ± 190 nm/s at 50% RH (*R* ^2^ = 0.9992). Such an almost 2-fold increase in the rate of indentation measured at 40% and 50% RH with 13.6 μm/s cantilever velocity is in sharp contrast with approximately similar rates of indentation measured at these two RH values using a lower cantilever velocity of 0.7 μm/s. Thus, consistent with our hypothesis, relatively slow cantilever velocity does in fact, limit accurate quantification of the rate of indentation, especially for relatively higher rates that are expected at elevated RH. Figure 2F shows the rate of indentation measured with 13.6 μm/s cantilever velocity versus RH, where a clear continuous increasing trend with increasing RH is observed, in contrast to the plateau observed at slower cantilever velocity (Figure 2C).

We next repeated the experiments shown in Figure 1B (red dots), collected at 0.7 μm/s, but now at a higher cantilever velocity of 13.6 μm/s, with results shown in Figure 1B, where blue dots represent average viscosity values at corresponding RH. As can be seen from Figure 1B, measured viscosity values at RH greater than ∼50% overlap well with Song et al.,^37^ in contrast with the red dots highlighted by the red oval that showed clear deviation. Thus, accurate viscosity quantification in the lower viscosity range is achieved using a higher cantilever velocity. We note, however, that the blue dots data shown in Figure 1B at RH below 45% appear to show systematically lower values than expected values reported by Song et al., although still within the reported ±10% uncertainty range. The origin of this deviation is likely due to indentation measurements utilizing relatively fast cantilever velocities over semisolid particles, which results in induced plastic-like deformation at the point of contact between the AFM tip and the particle. This leads the particle surface at the tip-particle interface to progressively shift more toward a less solid and more semisolid and liquid-like phase state. Thus, measured viscosity becomes lower because of indentation measurements than what it would be for the unperturbed particle. Previous AFM studies have demonstrated that increasing the indentation rate enhances the sensitivity of solids to viscous dissipation, revealing less semisolid and more liquid-like behavior that may not be apparent under slower indentation rate conditions.^30–32^ For example, nanorheological measurements of biological cells have shown that higher indentation frequencies (correspondingly higher indentation rates) or faster force–distance measurements lead to an apparent softening and increased viscous fluidity of the measured cell.^32^ We note that the extent of this deviation diminishes significantly as particle viscosity decreases, as can be clearly seen from the data shown in Figure 1B, leading to almost perfect overlap with Song et al. at elevated RH greater than 50%.

We next investigated how measured viscosities at two selected RH values change as a function of cantilever velocity. Figure 3 shows plots of measured viscosity for an individual submicrometer glucose particle at 40% RH (A) and 50% RH (B) as a function of the corresponding cantilever velocity. These RH values were selected for comparison because the cantilever velocity of 0.7 μm/s enables accurate viscosity measurement at 40% RH, whereas 13.6 μm/s yields accurate measurement at 50% RH (see Figure 1). As shown in Figure 3A, measured viscosity values using cantilever velocities at and below 7 μm/s fall within the uncertainty range of Song et al.,^37^ with the deviations beginning to emerge at velocities above 7 μm/s (shown by the red oval in Figure 3A). As discussed above, the origin of this deviation is due to AFM tip induced plastic-like deformation at the tip-particle interface, which yields lower measured viscosity. On the other hand, Figure 3B shows how measured viscosity at 50% RH begins to deviate from the expected range at velocities at and below 3 μm/s (indicated by the red oval), and the measured viscosities fall within the expected range at and above 5 μm/s. As discussed above, the origin of this deviation for the lower velocities is due to underestimation of the rate of indentation. Thus, based on these observations the following criteria can be established for the data set shown in Figure 3. For accurate quantification of individual glucose particle viscosities greater than ∼10^4^ Pa s, a lower cantilever velocity (≤7 μm/s) should be used, while for glucose viscosities less than ∼10^4^ Pa s, a higher velocity (5 μm/s–20 μm/s) should be used. We note, however, that these criteria are specific to glucose and may not be applicable for a sample with unknown viscosity. Next, we propose and discuss an iterative cantilever velocity framework that we established and validated, which enables identification of appropriate cantilever velocity required to accurately quantify single particle viscosity.

**Figure 3:**
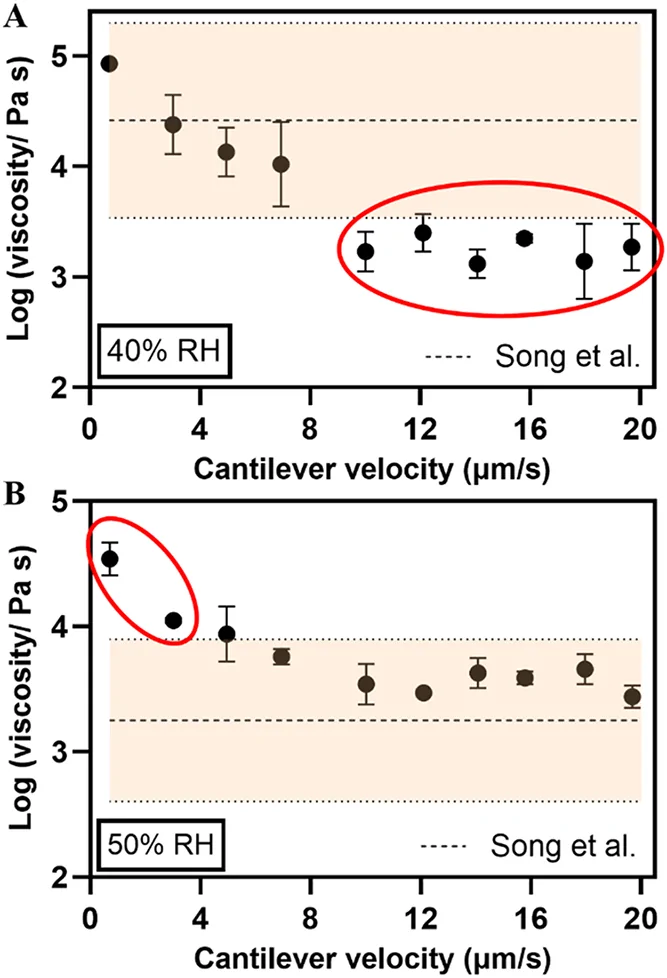
Plots of viscosity at 40% RH (A) and 50% RH (B) determined for an individual submicrometer glucose particle as a function of cantilever velocity. Dots are average values, and error bars represent one standard deviation. For some viscosity data points, the standard deviation is smaller than the size of the symbol. The black dashed lines correspond to the expected values from Song et al.,^37^ where the pink shaded area represents ±10% uncertainty. Red ovals indicate data points that deviate beyond ±10% uncertainty range shown by the pink areas.

Based on the cantilever velocity dependent AFM results shown in Figures 1–3, the following iterative framework (see Figure 4) is proposed to identify which cantilever velocity should be used for accurate viscosity quantification of an individual particle of unknown composition. The procedure begins by performing multiple force vs indentation measurements on an individual particle at a particular low RH (e.g., 20% RH) using various cantilever velocities (e.g., *n* = 4 with *v*_1_ = 0.7 μm/s, *v*_2_ = 7 μm/s, *v*_3_ = 14 μm/s, *v*_4_ = 20 μm/s). At this RH, the rate of indentation is determined for each cantilever velocity. Then these measurements are repeated using multiple velocities at several selected elevated RH (e.g., 5–10% increments between each RH value), followed by quantifying the corresponding rate of indentation. The framework then compares the measured rate of indentation at elevated RH vs lower RH (e.g., 30% vs 20% RH) at a particular cantilever velocity. Based on multiple experiments with various model systems, the following criterion is proposed to assess if the rate of indentation is increasing or reaching a plateau with increasing RH. For a particular lowest velocity (e.g., 0.7 μm/s), if the measured rate of indentation at elevated RH is more than 3% larger than that measured at lower RH, then the determined rate of indentation at this velocity is assumed to be accurate, and the corresponding force vs indentation data at this velocity should be fit to eq 1 to yield an accurate single particle viscosity result. On the other hand, if the two rates of indentation measured at a particular lowest velocity are within 3% of each other, then this cantilever velocity likely leads to inaccurate determination of rate of indentation, and should not be used; instead, the second lowest cantilever velocity data is used next, following the same comparison procedure. This iterative selection continues at each elevated RH until the appropriate velocity is identified across the full RH range, ensuring the rate of indentation shows the expected monotonic increase with RH. We note that one possible limitation of the proposed framework approach is if the particle viscosity does not change significantly with varying RH, e.g., an effloresced particle. We recommend equilibrating the sample prior to viscosity measurements at ∼5% RH (i.e., below the efflorescence point for most systems) for approximately 10–15 min to ensure majority of the particles are effloresced. We next show successful application of this framework to accurately quantify viscosities of glucose and sucrose submicrometer individual particles.

**Figure 4:**
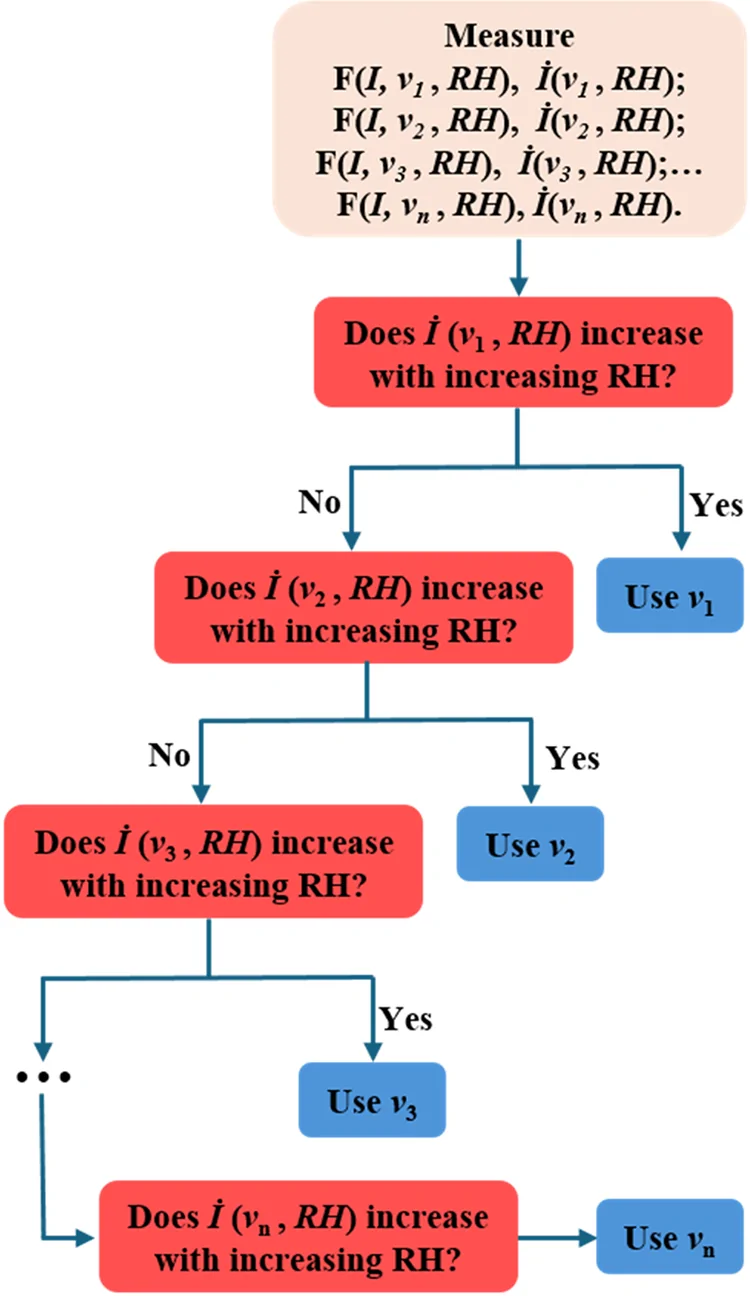
Classification iterative cantilever velocity framework to identify appropriate cantilever velocity for accurate quantification of single particle viscosity at a particular RH.

Figure 1C shows AFM quantified viscosity of individual submicrometer glucose particles as a function of RH over the 20–60% RH range that was obtained using the outlined framework above. Red and blue data points correspond to measurements obtained using two different cantilever velocities and the framework was utilized to determine which data set to use at a specific RH. Starting from 20% RH, with 0.7 μm/s velocity, the rate of indentation was increasing monotonically in 5% RH steps until 40% RH (Figure 2C). When RH increased from 40% to 45%, the relative increase in the rate of indentation measured was less than 3%, indicating that this cantilever velocity does not enable accurate quantification of the rate of indentation. Thus, according to the framework, measurements at higher cantilever velocity are needed. For this reason, a higher cantilever velocity of 13.6 μm/s was used for AFM measurements at and above 45% RH. As shown in Figure 2F, the rate of indentation was monotonically increasing with a relative increase greater than 3% between 45% RH and maximum RH of 60% used in this study. Thus, 13.6 μm/s cantilever velocity was used to quantify viscosity, with results shown in Figure 1C as blue dots. Collectively, the combined results demonstrate successful framework implementation and indicate that the AFM approach enables accurate quantification of viscosity within the range of ∼10^2.5^ to 10^7^ Pa s. Therefore, by identifying the appropriate cantilever velocity to accurately determine the rate of indentation, this framework enables an accurate and reliable viscosity quantification of an unknown particle across a wide RH range and correspondingly wide range of viscosities. In addition, the Young’s moduli for an individual glucose particle using both cantilever velocities across 20% – 60% RH show systematic reduction with increasing RH (Figure S2,A), consistent with increased water uptake progressively softening the particle.

To further validate the applicability of utilizing the varying cantilever velocity AFM measurements coupled with the iterative cantilever velocity framework toward accurate quantification of wider range of viscosities, an additional model system of single particle sucrose was studied. Figure 5A shows a representative AFM 3D height image at ∼30% RH of an individual sucrose particle (height of ca. 160 nm and base size of ca. 1100 nm), displaying rounded morphology consistent with previous studies,^42^ and the AFM determined contact angle between the particle and the underlying substrate of ∼66° (Figure S1). The red cross corresponds to an approximate center of the particle where AFM force measurements were performed. Figure 5B shows the average rate of indentation measured with 0.7 μm/s velocity versus the corresponding RH for an individual sucrose particle. As can be seen from the Figure 5B, the rate of indentation shows a monotonic relative increase greater than 3% with respect to increasing RH until 50% RH, while values at 55% and higher RH (indicated by the red oval) do not display the relative rate of indentation increase greater than 3%. Hence, in accordance with the framework, 0.7 μm/s velocity should only be used for data collected at and below 50% RH, and higher velocity is needed for RH at and greater than 55%. Thus, a higher cantilever velocity of 13.6 μm/s was utilized for RH at and greater than 55%, with corresponding measured rate of indention using this velocity shown in Figure 5C. As can be seen from Figure 5C, data points at RH at and greater than 55% show a clear continuous increasing trend with increasing RH. Figure 5D shows measured viscosity of individual sucrose particle versus RH, where red and blue data points correspond to AFM measurements obtained using 0.7 and 13.6 μm/s cantilever velocities, respectively. The combined viscosity results show excellent agreement with the expected values from Song et al.^37^ within the viscosity range of ∼10^2.9^ to 10^7^ Pa s. Additionally, fitted Young’s modulus values for individual submicrometer sucrose particle using both cantilever velocities across 37%–60% RH range show systematic reduction in the Young’s modulus with increasing RH (Figure S2,B), similar to the glucose results above and also consistent with our expectations.

**Figure 5:**
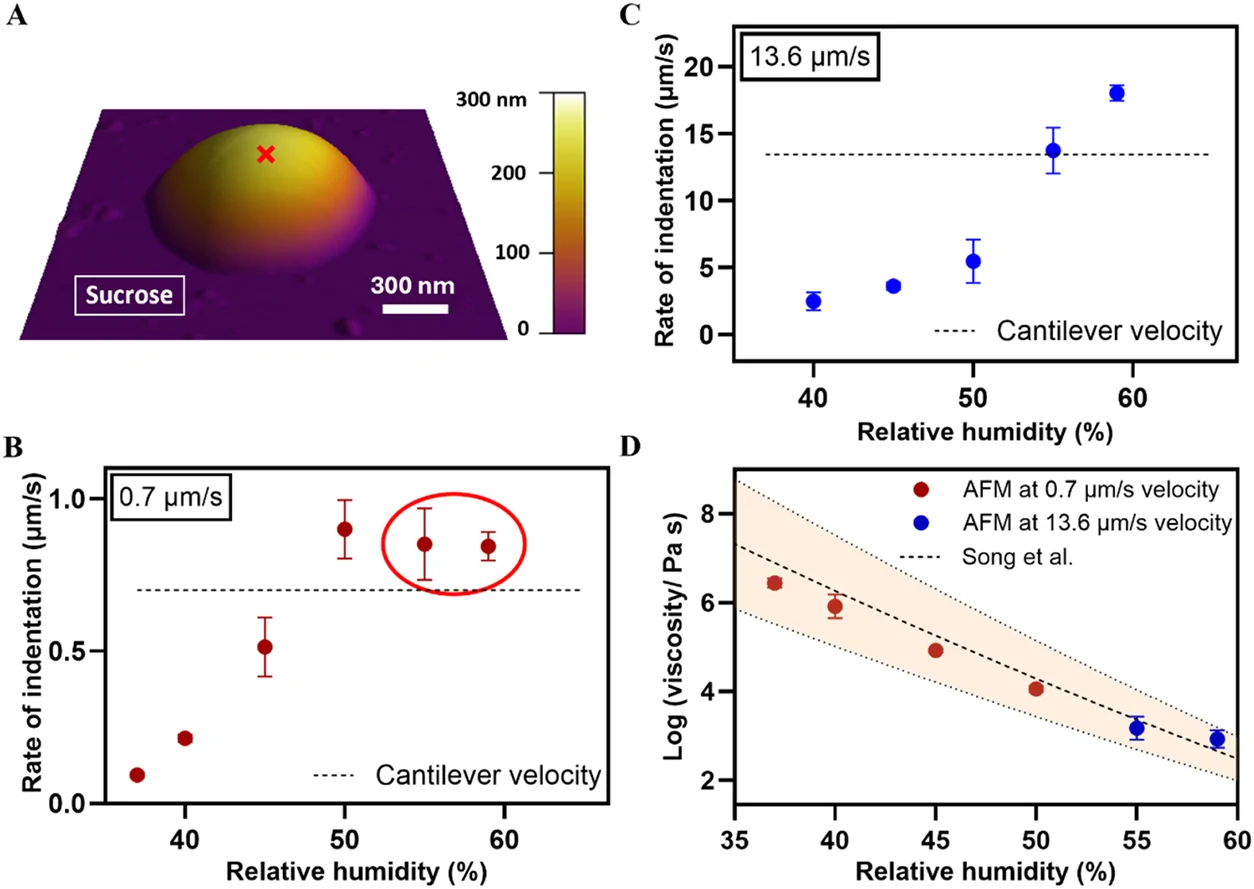
(A) Representative AFM 3D height image of a single sucrose particle at ∼ 30% RH displaying a rounded morphology (ca. 160 nm height and ca. 1100 nm base size). The red cross indicates where force measurements were performed. Average rate of indentation measured at 0.7 μm/s (B, red dots) and 13.6 μm/s (C, blue dots) cantilever velocity as a function of RH, where dashed lines indicate corresponding cantilever velocities. Red oval highlights data points deviating from the expected increasing trend with RH. (D) Combined average AFM viscosity data as a function of RH obtained using the iterative cantilever velocity framework. (red and blue dots for 0.7 μm/s and 13.6 μm/s velocities, respectively) Black dashed line indicates the expected values from Song et al.,^37^ where the pink shaded area represents ±10% uncertainty.

To further assess the effects of varying cantilever velocity on measured viscosity, viscosity was measured over an individual submicrometer sucrose particle at 50% RH with varying cantilever velocities ranging from 0.7 μm/s to 19.7 μm/s (Figure S3,A), where all viscosities were determined to be within the expected range. However, measured viscosities using cantilever velocities at or greater than 3.0 μm/s were systematically lower than expected, consistent with our previous observations for glucose that AFM tip induced plastic-like deformation at the tip-particle interface leads to an apparent lowering of the measured viscosity. On the other hand, viscosity versus velocity measurements were also performed over an individual submicrometer sucrose particle at an elevated RH of 56% RH (Figure S2,B), where results show that only the viscosity measured using 0.7 μm/s velocity was not within the expected range, and all other viscosity measurements at and above 3.0 μm/s cantilever velocities were within the expected viscosity range. This result is also consistent with our previous observations for glucose, where the origin of the deviation for lower velocity (0.7 μm/s in this case) is due to underestimation of the rate of indentation, which leads to the higher-than-expected measured viscosity. Collectively, the results of the viscosity quantification based on AFM force and indentation rate measurements at varying cantilever velocities on both glucose and sucrose single particle model systems coupled with the developed iterative cantilever velocity framework, indicate the applicability of AFM viscosity measurements of unknown submicrometer individual particles with accurate viscosity quantification within ∼10^2.5^–10^7^ Pa s, which covers a significant portion of the semisolid phase.

### Application of the AFM Viscoelastic Method for sub-100 nm Individual Semisolid Aerosols

Given the large number of atmospheric aerosols that are sub-100 nm in size,^45^ we next examined the possibility of utilizing the AFM method to quantify the viscosity of individual sub-100 nm particles collected from MOUDI stages 9 and 10, corresponding to aerodynamic diameters ranging from 0.18 to 0.055 μm. We note that prior to the present work, this AFM method had been applied only to submicrometer-sized particles. As a model system, sub-100 nm glucose nanoparticles were studied. AFM height imaging (Figure 6A) revealed that glucose nanoparticles exhibit rounded morphologies, same as those observed for submicrometer glucose particles (see Figure 1A). To test the applicability of the AFM viscoelastic method, AFM measurements and viscosity determinations were performed at two selected RH (35% and 50%) over 15 individual glucose nanoparticles with heights ranging from ∼20 to 100 nm and corresponding base sizes (area equivalent diameters) ranging from ca. 300 to 1500 nm. Specifically, viscosity was calculated using the data obtained with 0.7 μm/s cantilever velocity at 35% RH, while data collected with 13.6 μm/s velocity was used at 50% RH. Different cantilever velocities were utilized at these two RH values based on the viscosity results on submicrometer glucose particle shown in Figure 1C. Figure 6B,C shows AFM quantified viscosity of individual sub-100 nm glucose nanoparticles measured at 35% (Figure 6B) and 50% RH (Figure 6C) as a function of corresponding particle height, along with the expected viscosity results from Song et al.^37^ (see Figure S4 for similar plots but using volume equivalent diameters instead of particle heights). The measurements and data analysis to quantify nanoparticles’ viscosity were identical to that for submicrometer particles described above, except for one difference. To minimize the contribution from the underlying stiff substrate (Si wafer for the present study) to the measured force as the AFM tip indents into the nanoparticle, for nanoparticles with heights less than ∼40 nm, the maximum indentation distance was limited to less than 25% of the particle height, while previously for submicrometer particles the maximum indentation of 10 nm was used. We note, assuming the lowest indentation distance required to obtain a good fit for the viscosity is 5 nm, the practical limitation on the smallest nanoparticle height amendable for the accurate measurements is ∼20 nm. Two important conclusions can be drawn from the data shown in Figure 6B,C. First, no apparent size-dependent viscosity trends are observed within the studied size range, with results largely overlapping with those measured on submicrometer glucose and the expected literature values. We note that, while no size-dependent trends were present for the model glucose nanoparticles system, the overall approach nonetheless can be similarly used to examine possible size-dependent trends in aerosol viscosity, especially when applied to real atmospheric aerosols. Second, viscosity measurements of individual glucose nanoparticles are in excellent agreement with expected literature results, confirming the applicability of the AFM viscoelastic method for accurately quantifying the viscosity of individual semisolid nanoparticles with heights as low as ∼20 nm.

**Figure 6:**
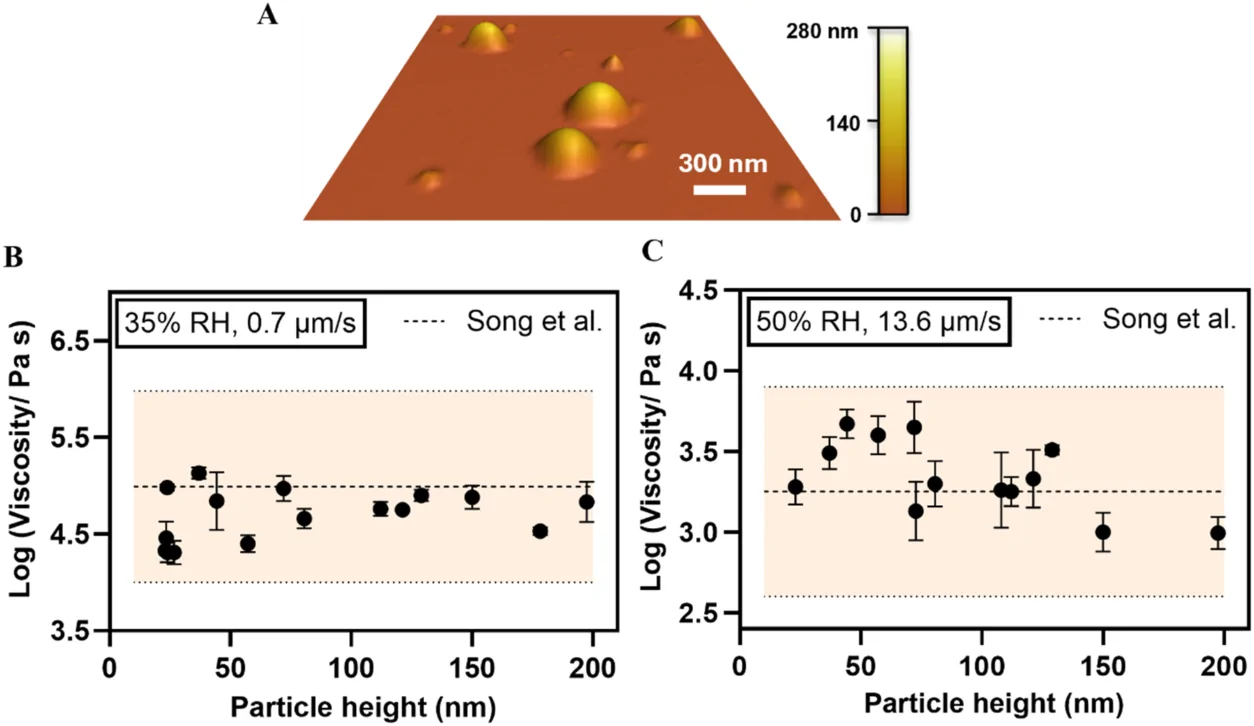
(A) Representative AFM 3D height image of glucose nanoparticles at ∼30% RH displaying rounded morphologies. Viscosity of individual glucose nanoparticles as a function of particle height at 35% RH (B, 0.7 μm/s) and 50% RH (C, 13.6 μm/s). Dots are average values, and error bars represent one standard deviation. The black dashed lines correspond to the expected values from Song et al.,^37^ where the pink shaded area represents ± 10% uncertainty.

## Conclusions

In this work, we develop a new experimental AFM approach based on force–indentation distance and indentation rate measurements using varying cantilever velocities to quantify viscosity of individual substrate-deposited semisolid aerosol particles. Using submicrometer individual glucose and sucrose particles as model systems, we demonstrate how the experimental approach enables accurate single particle viscosity quantification within ∼10^2^·^5^ to 10^7^ Pa s. We further develop an iterative cantilever velocity framework that enables the determination of the appropriate cantilever velocity required for accurate viscosity quantification of individual submicrometer semisolid aerosols without prior knowledge of their chemical identity. Furthermore, we show that the AFM method is also applicable for viscosity quantification of individual sub-100 nm aerosols, yielding accurate viscosity measurements for aerosols as low as 20 nm in height. By expanding both the accessible viscosity quantification range to progressively less semisolid phase state and expanding quantifiable particle size range to now include nanoscale, we anticipate this new cantilever velocity dependent AFM approach will be applied to a wide range of atmospheric aerosols from various sources and sizes, ultimately aiding a better understanding of the role and variability of viscosity of atmospheric aerosols and its impact on the climate and atmospheric processing.

## Supplementary Material



## Notes

Details of contact angle between glucose and sucrose submicrometer particles and underlying surfaces, Young’s modulus versus RH quantified from the AFM viscoelastic method for individual submicrometer glucose and sucrose particles, plots of AFM determined viscosity for an individual submicrometer sucrose particle as a function of cantilever velocity measured at 50% and 56% RH, plots of viscosity measured on individual glucose nanoparticles as a function volume equivalent diameter at 35% RH with 0.7 μm/s cantilever velocity and at 50% RH with 13.6 μm/s cantilever velocity (PDF)
