# Supplementary material for: Quantifying Viscosity of Semisolid Submicrometer and Nanosized Individual Aerosols Using Atomic Force Microscopy with Varying Cantilever Velocity
Source: Anal Chem. 2026 Oct 1;98(41):29471–80. doi: 10.1021/acs.analchem.6c02398 (PMC13689400; doi:10.1021/acs.analchem.6c02398)
Supplement: Supplementary file 1 [file ac6c02398_si_001.pdf]

Supporting Information

Quantifying Viscosity of Semisolid Submicrometer- and Nano-sized Individual Aerosols using Atomic Force Microscopy with Varying Cantilever Velocity

*Ruwini A. Weerasinghe, Chamika K. Madawala, Alexei V. Tivanski**

Department of Chemistry, University of Iowa, Iowa City, Iowa 52242, USA.

Corresponding author: Alexei V. Tivanski (alexei-tivanski@uiowa.edu)

Table of Contents

Figure S1. (contact angle between glucose and sucrose submicrometer particles and underlying surfaces) S2

Figure S2. (Young's modulus versus RH quantified from the AFM viscoelastic method for individual submicrometer glucose and sucrose particles) S3

Figure S3. (plots of AFM-determined viscosity for an individual submicrometer sucrose particle as a function of cantilever velocity measured at 50% and 56% RH)........ S4

Figure S4. (Plots of viscosity measured on individual glucose nanoparticles as a function of volume equivalent diameter at 35% RH with 0.7 μm/s cantilever velocity, and at 50% RH with 13.6 μm/s cantilever velocity) S5

References........ S5

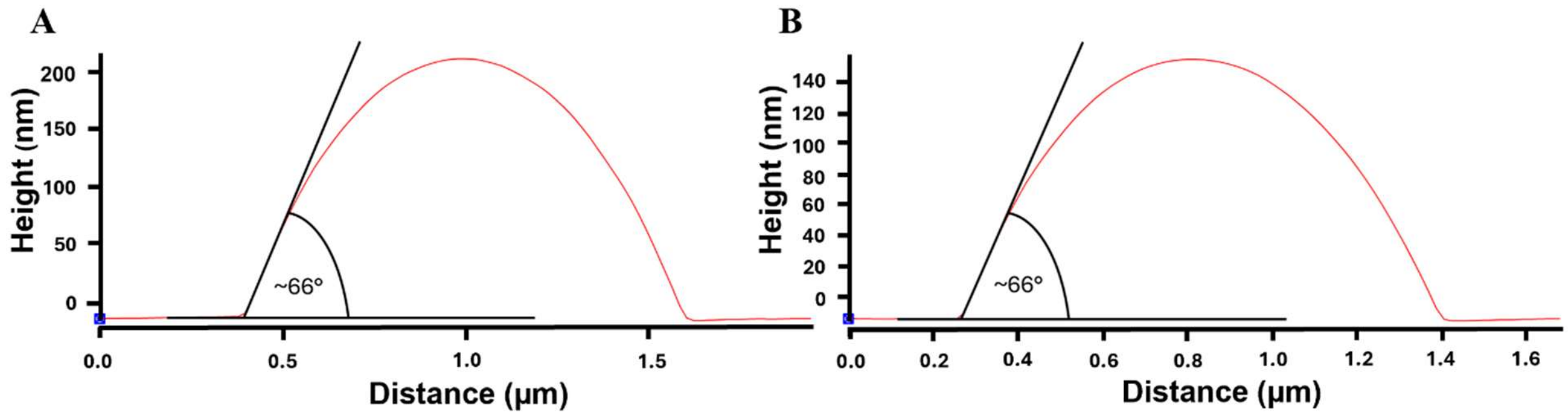

Figure S1. AFM cross-sectional height profiles over individual glucose (A) and sucrose (B) particles deposited on Rain-X coated silicon substrates at 30% RH. Solid lines and corresponding contact angle measurements reveal ~66° contact angle between each particle and the underlying substrate.

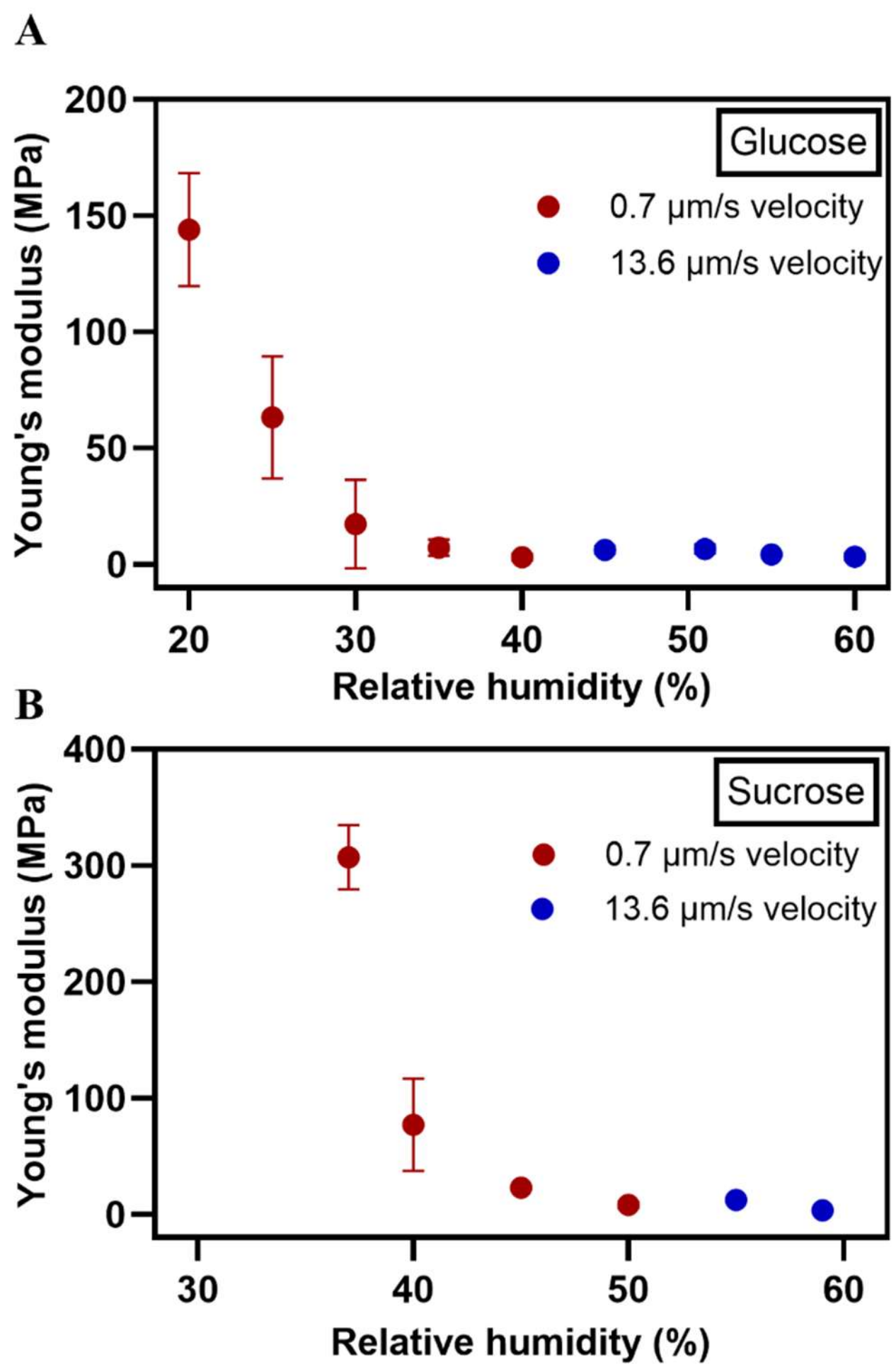

Figure S2. Young's modulus quantified from the AFM viscoelastic model with 0.7 μm/s (red dots) and 13.6 μm/s cantilever velocity (blue dots) as a function of RH for (A) glucose and (B) sucrose individual submicrometer particles. Here dots are average Young's modulus values, and error bars correspond to one standard deviation of the mean. For some Young's modulus data, the standard deviation is smaller than the size of the symbol.

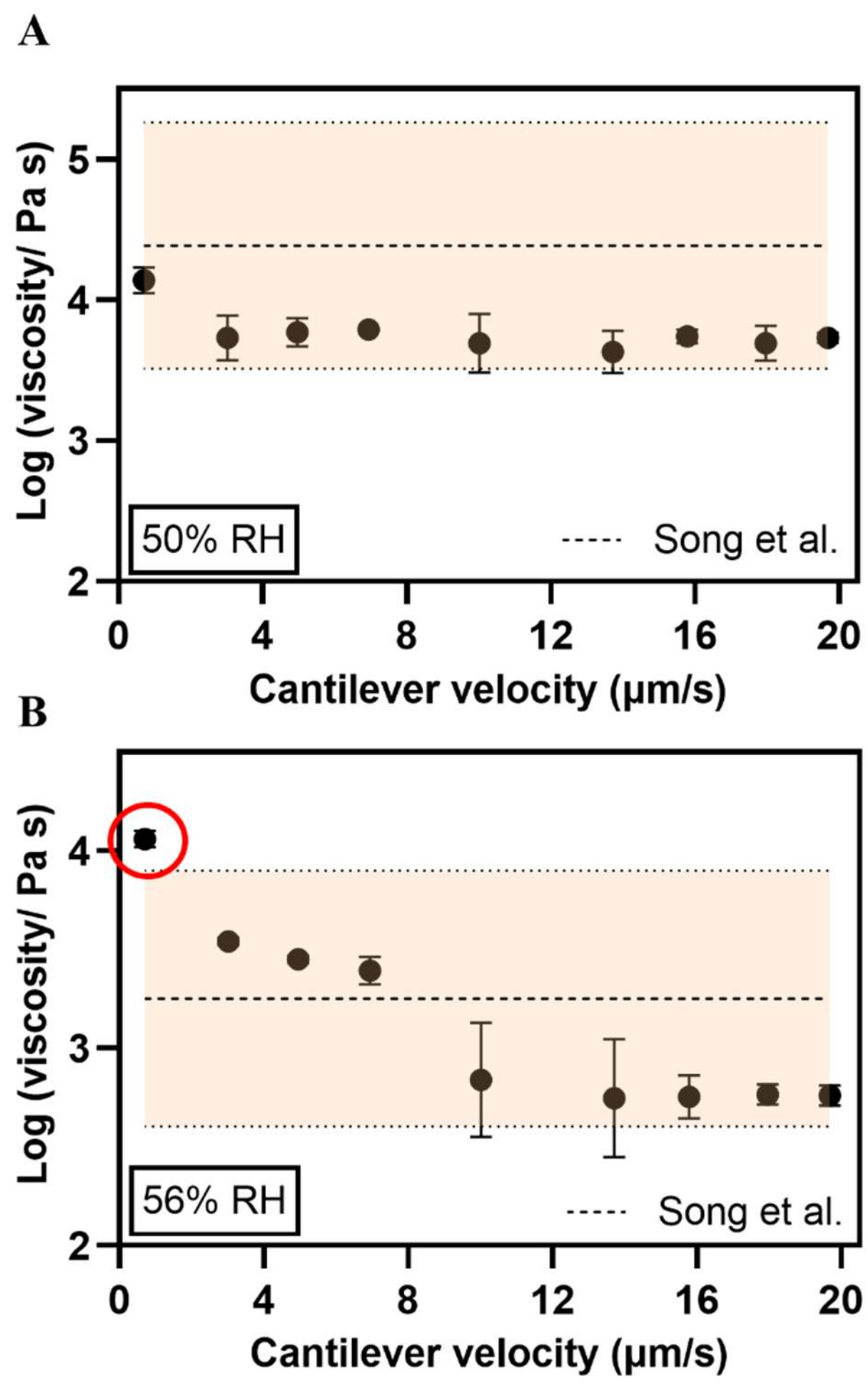

Figure S3. Plots of viscosity at 50% (A) and 56% RH (B) determined for an individual submicrometer sucrose particle as a function of cantilever velocity. Dots are averaged values, and error bars represent one standard deviation of the mean. For some viscosity data points, the standard deviation is smaller than the size of the symbol. The black dashed lines correspond to the expected values from Song et al.,[1] while the pink shaded area represents ±10% uncertainty in the expected values. Red oval indicates the viscosity data point that deviates beyond the expected ±10% uncertainty range.

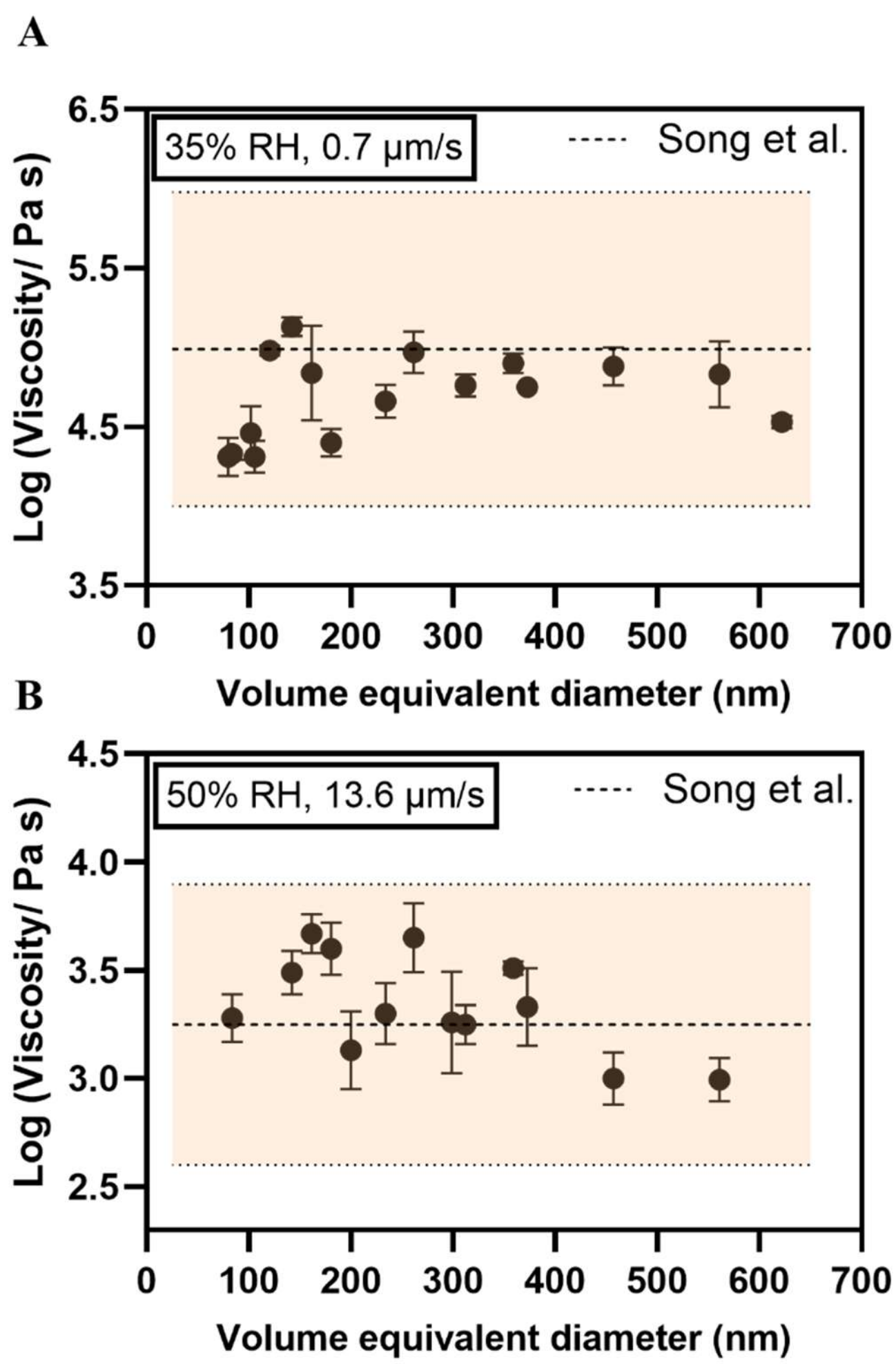

Figure S4. Plots of viscosity measured on individual glucose nanoparticles as a function of the corresponding volume equivalent diameter at 35% RH with 0.7 μm/s cantilever velocity (A) and at 50% RH with 13.6 μm/s cantilever velocity (B). Dots are average values, and error bars represent one standard deviation. For some viscosity data points, the standard deviation is smaller than the size of the symbol. The black dashed lines correspond to the expected values from Song et al.,[1] where the pink shaded area represents ±10% uncertainty.

References

(1) Song, Y. C.; Haddrell, A. E.; Bzdek, B. R.; Reid, J. P.; Bannan, T.; Topping, D. O.; Percival, C.; Cai, C. Measurements and Predictions of Binary Component Aerosol Particle Viscosity. *J. Phys. Chem. A* **2016**, *120* (41), 8123–8137.
